# Associations between physical activity intensity, frequency, and anxiety symptoms among South Korean adolescents: a sociodemographic subgroup analysis

**DOI:** 10.3389/fpubh.2026.1934776

**Published:** 2026-09-30

**Authors:** Jihyun Song, Wonseok Choi

**Affiliations:** 1 Keimyung Institute for International Studies, Keimyung University, Daegu, Republic of Korea; 2 Department of Kinesiology, Keimyung University, Daegu, Republic of Korea

**Keywords:** anxiety, high school students, middle school students, muscle-strengthening activities, physical activity intensity

## Abstract

**Background:**

Anxiety symptoms are an important public-health concern among South Korean adolescents. Although physical activity (PA) may support adolescent mental health, its associations with anxiety may differ according to activity type, intensity, frequency, and sociodemographic characteristics. Clarifying these differences could provide more inclusive school- and community-based health promotion. This study examined associations between PA types/intensities and frequencies and anxiety symptoms, and whether these associations differed by gender, school level, and socioeconomic status (SES).

**Methods:**

Using data from 54,653 adolescents in the 20th Korea Youth Risk Behavior Web-Based Survey, this study conducted a single-group structural equation modeling analysis to reveal the overall associations between types/intensities and frequencies of PA and anxiety symptoms. This study also conducted multi-group structural equation modeling analyses to test whether these associations differed across sociodemographic factors.

**Results:**

In the single-group SEM, all types/intensities of PA for 3 days or more per week were generally associated with lower anxiety compared to PA for 0–2 days (βs ranging from −0.021 to −0.057, *p* < 0.01). These patterns, however, varied significantly across sociodemographic factors, such as gender, school level, and socioeconomic status in the multi-group SEM. Specifically, vigorous PA was most consistently associated with lower anxiety across multiple subgroups, whereas muscle-strengthening activities were significantly protective primarily at high frequency (≥ 5 days/week) among high school students and low-SES adolescents. In contrast, moderate-to-vigorous PA for ≥ 5 days/week was unexpectedly associated with higher anxiety levels among females (*β* = 0.055, *p* = 0.033) and high-SES adolescents (*β* = 0.111, *p* = 0.011).

**Conclusion:**

More frequent PA appeared to be associated with fewer anxiety symptoms, although the associations were small and varied across sociodemographic groups. Because high-frequency Moderate-to-vigorous PA was positively linked to anxiety in certain subgroups, PA promotion should consider activity type, volume, and sociodemographic contexts rather than assuming uniform benefits. Education and public-health authorities should consider expanding equitable access to enjoyable, age-appropriate PA within comprehensive adolescent mental-health strategies, with program evaluation sensitive to gender, school level, and SES. Longitudinal and intervention studies are needed before anxiety-specific activity-frequency recommendations can be established.

## 1 Introduction

Adolescents are at risk of mental health problems such as anxiety symptoms involving negative feelings of excessive worry, fear, and avoidance (1, 2). This risk often increases the possibility of long-term adverse consequences, such as the same (homotypic continuity) and other symptoms or disorders (heterotypic continuity), substance dependence, suicidal behavior, and poor educational and developmental outcomes (3, 4). In particular, mental-health symptoms represent a critical public-health concern among Korean adolescents. The recent national data from the 2024 Korea Youth Risk Behavior Web-Based Survey (KYRBS) reported that Korean adolescents experienced diverse mental health challenges, with 27.7% reporting depressive symptoms, 18.8% chronic loneliness, 14.1% moderate-to-severe anxiety symptoms, and 12.7% suicidal ideation, and the prevalence of these challenges has increased (5). In particular, addressing anxiety symptoms is critical in that they serve a foundational role in the developmental trajectory of internalizing psychopathology. Recent longitudinal network and developmental psychopathology evidence has demonstrated that anxiety symptoms often function as temporal precursors and developmental gateways to subsequent mental/emotional and functional impairments, such as depression, stress, and burnout (6–8). Conducting cross-lagged panel network analyses, Wang et al. (7) established the robust predictive effects of anxiety on downstream mental health problems, while Zhang et al. (8) verified that anxiety operates as a critical catalyst within adolescent symptom structures. In addition, Lahey et al. (6), within a hierarchical causal framework, highlighted that anxiety initiates behavioral avoidance, which directly precipitates secondary depression and burnout. Therefore, understanding anxiety symptoms is essential not only because they represent the core component of internalizing problems, but also because intercepting these early anxiety trajectories prevents the widespread dissemination of psychiatric comorbidity.

To prevent or at least reduce such anxiety symptoms, scholars have highlighted engaging in physical activity (PA) as a modifiable factor through physiological adaptations (e.g., stabilizing stress-response systems, facilitating neurotransmitter activity) and psychological mechanisms (e.g., enhancing self-efficacy) (9–11). In other words, participating in PA may help adolescents reduce anxiety symptoms by making their brain more resilient to stress, optimizing euphoric states with reduced pain, and developing a stronger sense of their ability to succeed. In fact, the inverse association between PA and anxiety symptoms has been well documented in the literature (12–16). In review articles, for example, Biddle and Asare (12) found a small but beneficial effect of PA on anxiety symptoms for young people despite the limited number of reviews (*n* = 4). Similarly, McDowell et al. (13), in a meta-analysis of prospective studies with at least a 1-year follow-up, reported a moderate longitudinal effect of PA on lower anxiety symptoms. These research findings suggest that PA is likely to play a critical role in addressing anxiety symptoms among adolescents.

With the growing empirical evidence of the inverse association, researchers have attempted to determine potential types/intensities and frequencies of PA that can be effectively applied to program design for lower anxiety symptoms (13, 14, 17–19). Categorizing types/intensities of PA into moderate PA (MPA), vigorous PA (VPA), and muscle-strengthening activities (MSA), Shin and Lee (19) revealed that anxiety symptoms were significantly higher in the absence of performing VPA and MSA, respectively, among 117,351 Korean adolescents. Combining MPA and VPA, some scholars have found that more frequent engagement in moderate-to-vigorous PA (MVPA) was associated with lower anxiety symptoms than less frequent engagement (9, 10, 14, 16). McMahon et al. (14), for example, demonstrated that participating in MVPA for 4–7 days in the past two weeks was negatively associated with anxiety symptoms compared to performing MVPA for ≤ 3 days among 11,110 adolescents across 10 European countries. Similarly, Murphy et al. (18) revealed that MPVA for ≥ 4 days in the past 14 days was negatively associated with anxiety symptoms compared to MVPA for ≤ 3 days among 5,661 Irish adolescents. Building upon these findings, previous studies suggest that the more adolescents participate in any type of PA more frequently, the more likely it is to correlate with lower anxiety symptoms.

However, these general benefits of frequent PA participation for lower anxiety symptoms may not apply uniformly to all individuals. Empirical evidence has demonstrated that the associations of PA types/intensities and frequencies with anxiety symptoms are often shaped by sociodemographic characteristics, such as gender, school levels, and socioeconomic strata (2, 10, 15, 19, 20). These subgroups have frequently indicated distinct behavioral patterns and symptom manifestations, with some demonstrating pronounced benefits while others display attenuated or even unexpected patterns. For example, for gender, Asztalos et al. (20) demonstrated that while men exhibited anxiety reduction and symptom relief from VPA, women experienced such mental health benefits primarily through MPA and walking. Tian et al. (15) identified a nuanced moderating role of gender, reporting an unexpected direct pattern among females. In the study, high-intensity PA exerted a direct positive effect on anxiety among female students, implying that VPA and MSA may act as an acute physical stressor or heighten anxiety sensitivity in females. For school levels, previous studies observed progressive increases in baseline stress and anxiety disorder scores as adolescents transition from middle school to high school (2, 10). For SES, Humbert et al. (21) found that low-SES youth have less PA participation than their high-SES counterparts because of facility costs, safety concerns, and a lack of adult supervision. Crucially, when low-SES adolescents do engage in frequent VPA at least 5 days per week, Kim et al. (10) revealed that its protective effects are particularly pronounced among low-SES youth. Taken together, these findings confirm that the associations between PA and anxiety symptoms are not universal, but can be moderated by gender, school levels, and SES.

Despite these valuable insights, several critical gaps remain in the existing literature regarding the associations between adolescent PA and anxiety symptoms. While recent studies among Korean youth have evaluated PA in relation to anxiety symptoms, they have predominantly relied on aggregated or binary PA indicators without fully differentiating the simultaneous impacts of both PA types/intensities and frequencies (4, 10, 16). Specifically, prior national investigations often treated PA measures as a single composite variable or focused on isolated intensity levels, leaving it unclear how distinct PA types/intensities and their potential weekly frequency thresholds differentially influence anxiety symptoms. In addition, although sociodemographic disparities in adolescent anxiety symptoms are well-acknowledged, few studies have systematically evaluated how the protective associations of specific PA types/intensities and frequency thresholds vary simultaneously across gender, school levels, and socioeconomic strata within a single unified framework.

Building on previous research evidence articulated above, the purpose of this study was to determine the associations between types/intensities and frequencies of PA and anxiety symptoms across sociodemographic factors among Korean adolescents. To fulfill the purpose, two specific research questions guided the study: (a) How do distinct combinations of PA types/intensities and frequencies associate with adolescent anxiety symptoms? (b) Do gender, school level, and SES significantly moderate these associations? These research questions were addressed by testing two hypotheses: (a) the associations between combinations of PA types/intensities and frequencies would vary (Hypothesis 1), and (b) these associations would differ across gender, school level, and SES (Hypothesis 2). Figures 1, 2 display the hypothesized *a priori* models to be tested in this study. The findings of this study would guide the development of effective PA programs for adolescent anxiety, providing potential frequency thresholds of PA.

**Figure 1:**
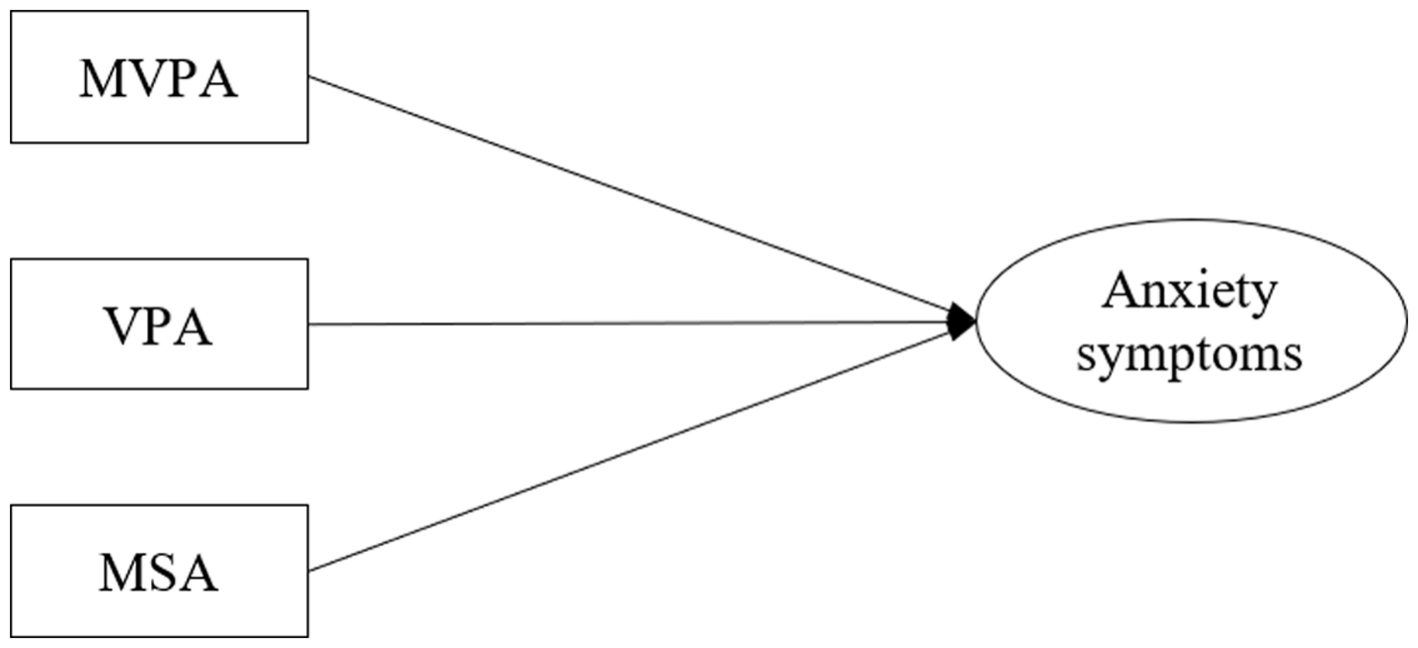
Hypothesized model of the association between PA and anxiety symptoms.

**Figure 2:**
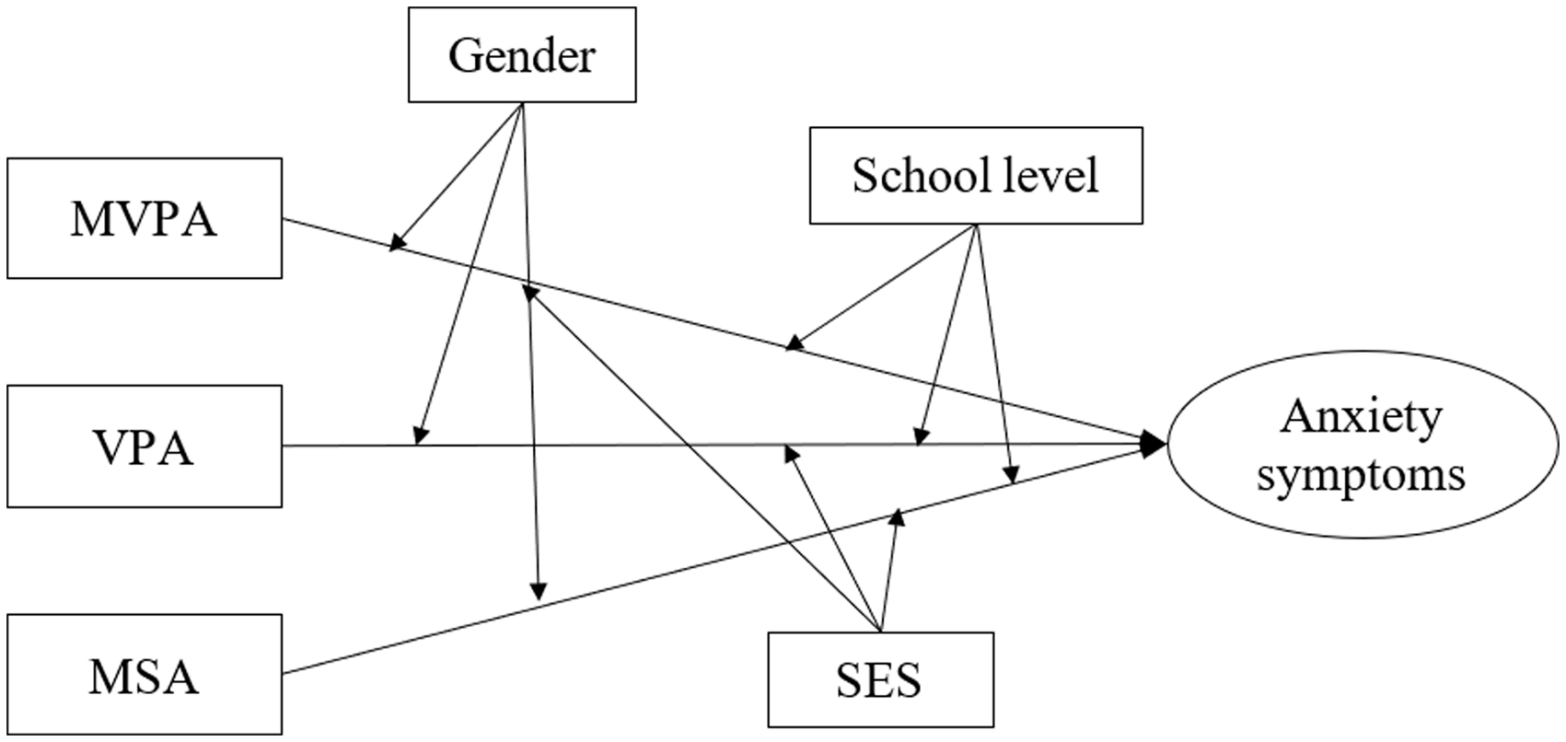
Hypothesized model of the moderating role of sociodemographic factors in the association between PA and anxiety symptoms.

## 2 Materials and methods

### 2.1 Participants

The present study used data from the 20th KYRBS conducted by the Korea Disease Control and Prevention Agency (KDCA), a national surveillance system benchmarked against the Youth Risk Behavior Surveillance System (YRBSS) of the US Centers for Disease Control and Prevention (CDC), in which Korean middle and high school students reported health-related indicators, such as PA, dietary behaviors, and mental health, between June 10 and July 6, 2024 (22). The target population was defined based on the Ministry of Education’s directory of middle and high schools, excluding specialized schools, such as special education and alternative schools (5). The sample was selected employing a stratified multi-stage cluster sampling method following the procedures: (a) the population of schools was stratified into 117 strata based on 39 regional clusters (classified by administrative metropolitan cities/provinces) and school types (middle, general high, and vocational high schools); (b) the sample was allocated equally between middle and high schools. The number of sample schools (400 middle schools and 400 high schools) was determined based on city size, school type (boys-only, girls-only, co-educational), and school division (e.g., general, specialized). Sampled schools with fewer than 50 students, incomplete grade levels, or scheduled closures were replaced with similar schools within the same stratum. Across the 800 sampled schools, one class per grade was selected from each school, resulting in three sample classes per school (2,400 classes in total). All students enrolled in the sampled classes were eligible, potential participants; but those with long-term absences, severe disabilities unable to respond independently, and literacy impairments were excluded as per KDCA survey protocols. Of the 800 sample schools, 799 participated (99.9%), and of 57,580 eligible students, 54,653 completed the survey (94.9% response rate; 96.1% for middle school, 93.6% for high school). A detailed comparison of demographic characteristics between the sample and the national adolescent population is presented in Table 1. Among the 54,653 student responses, missing values on the variables used in this study (*n* = 1,487, 2.7%) were handled using multiple imputation through fully conditional specification, generating 30 imputed datasets (23). The imputation model included all analysis variables used in this study.

**Table 1:** Comparison of study sample demographics with the national population.

Characteristic	National population, *N* (%)	KYRBS sample, *n* (%)
Total students	2,612,077 (100.0%)	54,653 (100.0%)
School level		
Middle school	1,293,048 (49.5%)	29,087 (53.2%)
High school	1,319,029 (50.5%)	25,566 (46.8%)
Gender		
Male	~1,348,000 (~51.6%)	28,090 (51.4%)
Female	~1,264,000 (~48.4%)	26,563 (48.6%)
Number of schools	5,673 (100.0%)	799 (Participating)

KYRBS, Korea Youth Risk Behavior Web-Based Survey; *N*, total population size; *n*, sample size; %, percentage.

### 2.2 Measures

#### 2.2.1 Physical activity

The KYRBS includes three types/intensities of PAs: MVPA, VPA, and MSA. This classification of the PA types/intensities is based on public health guidelines and developmental literature, allowing for the independent and combined evaluation of aerobic activity intensities and muscle-strengthening behaviors, which target distinct physiological and mental health outcomes in adolescents (7, 11, 16, 19). The question about MVPA was, “During the past 7 days, how many days did you participate in PA (regardless of its type) where your heart rate increased, or your breath was heavy for more than 60 min per day?” The question about VPA was, “During the past 7 days, how many days did you participate in vigorous PA that made you out of breath or sweating?” The question about muscular exercise was, “During the past 7 days, how many days did you participate in muscle-strengthening activities, such as push-ups, sit-ups, weightlifting, dumbbell exercises, pull-up bar exercises, parallel bar exercises, etc.?” Responses were then recorded on an 8-point scale, ranging from 1 (0 days) to 8 (7 days). Based on the literature (13, 24), participation frequencies for each type/intensity of PA were grouped into “0–2 days,” “3–4 days,” and “≥ 5 days.” This classification was applied to maintain operational consistency across all activity domains and to ensure model stability in hypothesis testing.

#### 2.2.2 Anxiety symptoms

Anxiety symptoms were measured using the Generalized Anxiety Disorder-7 (GAD-7), consisting of seven items (5). One example question is, “Over the last two weeks, how often have you been bothered by the following problems?” with a response item, “Worrying too much about different things.” Responses were rated on a 4-point scale (1 = Not at all, 4 = Nearly every day). Responses were averaged to represent overall patterns for descriptive purposes. All seven items were modeled as observed indicators of the latent structure of the variable, whereas all seven items were modeled as observed indicators of the latent anxiety variable for hypothesis testing in structural equation modeling. The Korean version of the GAD-7 has demonstrated high internal consistency (Cronbach’s *α* = 0.915) and convergent validity (*r* = 0.756) in Korean validation research (25) and has also shown high internal consistency (Cronbach’s *α* = 0.93) among Korean adolescents (26).

#### 2.2.3 Sociodemographic factors

Sociodemographic factors included gender (i.e., female and male), school level (i.e., middle and high school), and SES (i.e., high, medium, and low SES).

### 2.3 Data reduction and analysis

The variables used in this study comprised three types/intensities of PA (i.e., MVPA, VPA, and MSA) as exogenous, ordered variables; anxiety symptoms as endogenous, ordered variables; and sociodemographic factors as binary (i.e., gender, school level) or ordinal (i.e., SES) moderators. For PA frequency, two dummy variables were created to compare PA for 3–4 days and ≥ 5 days with the reference category of PA for 0–2 days. SES was also dummy-coded into two subgroups (i.e., middle and high SES) with low SES as a reference category. Descriptive analyses were conducted to display the data distribution patterns. The scores for anxiety symptoms were averaged for descriptive purposes prior to creating a latent variable for further analyses. Given the nature of the ordinal variables (e.g., anxiety symptoms), Spearman’s correlation analysis was performed as a preliminary step.

For Hypothesis 1, a single-group structural equation modeling (SEM) was used to analyze the overall association between the types/intensities and frequencies of PA and anxiety symptoms, without any sociodemographic covariates/moderators. For Hypothesis 2, multi-group SEM was employed to examine the role of sociodemographic factors (i.e., gender, school level, and SES) in moderating the relationship between PA and anxiety symptoms. When one factor served as a moderator, the others were included as covariates. Specifically, when treating a specific demographic factor as a grouping moderator, the other two were simultaneously included and controlled as covariates in the model. Gender and school level were binary-coded, with males and middle school students as reference groups, respectively. SES was also dummy-coded into middle- and high-SES groups, with low SES as the reference group, when included as a covariate in the gender- and school-level models, whereas it was categorized into three distinct subgroups (low, middle, and high SES) to serve as a grouping moderator in the SES-moderated multi-group models. Prior to testing structural paths across sociodemographic groups, a series of multi-group CFAs was conducted to assess the measurement invariance of anxiety using configural (no equality constraints), metric (factor loadings constrained equal), and scalar (factor loadings and item intercepts constrained equal) models. Establishing scalar invariance supports valid comparisons of latent constructs across groups. Measurement invariance was evaluated as established if ΔCFI ≤ 0.01 and ΔRMSEA ≤ 0.015, thereby ensuring valid comparisons of latent constructs across groups (27). In addition, pairwise multi-group CFAs across SES levels (high vs. middle, high vs. low, and middle vs. low) were conducted to identify potential subgroup-specific non-invariance. After establishing scalar invariance, group differences in structural paths from PA to anxiety were tested by comparing (a) a baseline multi-group structural model in which the paths were freely estimated across groups with (b) a constrained model in which the paths were held equal. Differences in structural paths were evaluated using Satorra–Bentler scaled (mean-adjusted) Δχ^2^_scaled_ tests (28).

Weighted least squares with mean and variance adjustment (WLSMV) was used to account for the ordinal nature of anxiety indicators. This approach is appropriate when SEM models include ordinal endogenous variables with a large sample size (≥ 500) (29). Analyses were performed on unweighted data because the primary sampling stratification characteristics (i.e., gender, school level, and SES) were explicitly incorporated as covariates and moderating subgroups, satisfying conditional noninformative selection and yielding unbiased structural estimates (30). Satorra–Bentler scaled fit statistics were reported to evaluate overall model adequacy under WLSMV estimation. This method can prevent potential fit distortions associated with standard maximum likelihood estimation, including the scaled chi-square (χ^2^_scaled_), scaled comparative fit index (CFI_scaled_), scaled Tucker–Lewis index (TLI_scaled_), scaled root mean square error of approximation (RMSEA_scaled_) with its 90% CI, and standardized root mean square residual (SRMR) (29). Although conventional cutoff criteria were originally derived under maximum likelihood estimation for continuous data (31), and fit indices under WLSMV estimation for ordinal data can be sensitive to sample size and model characteristics (32), the widely accepted benchmarks (CFI_scaled_/TLI_scaled_ ≥ 0.95, RMSEA_scaled_ ≤ 0.06, and SRMR_scaled_ ≤ 0.08) were applied as practical benchmarks (31). All coefficients for the associations were reported as standardized regression coefficients (*β*). All the analyses were conducted using the lavaan package in R (version 4.5.2).

## 3 Results

Table 2 describes descriptive statistics for all measured variables in this study. Of a total of 54,563 students, most were insufficiently engaged in MVPA, VPA, and MSA (0–2 days/week), ranging from 31,447 (57.5%) to 43,822 (80.2%), while only a small number reported engaging in these types/intensities of PA for ≥ 5 days, ranging from 3,676 (6.7%) to 10,180 (18.6%). The students demonstrated a low level of anxiety symptoms (*M* = 1.64, *SD* = 0.68). Table 3 displays Spearman’s correlation coefficients for the variables, ranging from *r* = −0.27 to 0.65, and all were statistically significant (*p* < 0.001), providing a preliminary foundation for subsequent analyses.

**Table 2:** Descriptive statistics of the measured variables.

Variable	*n* (%)
MVPA	
0–2 days	32,794 (60.0%)
3–4 days	12,072 (22.1%)
≥ 5 days	9,787 (17.9%)
VPA	
0–2 days	31,447 (57.5%)
3–4 days	13,026 (23.8%)
≥ 5 days	10,180 (18.6%)
MSA	
0–2 days	43,822 (80.2%)
3–4 days	7,155 (13.1%)
≥ 5 days	3,676 (6.7%)
Anxiety	1.64 (0.68)
Gender	
Male	28,090 (51.4%)
Female	26,563 (48.6%)
School level	
Middle school	29,087 (53.2%)
High school	25,566 (46.8%)
SES	
High SES	23,143 (42.3%)
Middle SES	30,415 (55.7%)
Low SES	1,095 (2.0%)

Values are presented as *n* (%) except for anxiety symptoms, which were reported as mean (standard deviation) based on the average GAD-7 score for descriptive purposes. MVPA, moderate-to-vigorous physical activity; VPA, vigorous physical activity; MSA, muscle-strengthening activities; SES, socioeconomic status; GAD-7, Generalized Anxiety Disorder 7-item scale.

**Table 3:** Descriptive statistics and Spearman’s correlation.

Variable	1	2	3	4	5	6
1. MVPA						
2. VPA	0.65					
3. MSA	0.35	0.37				
4. Anxiety	−0.08	−0.09	−0.07			
5. Gender	−0.27	−0.27	−0.25	0.21		
6. School level	−0.15	−0.20	−0.03	0.03	0.01	
7. SES	−0.05	−0.05	−0.02	0.07	0.04	0.10

All correlation coefficients are significant at the *p* < 0.001 level.

MVPA, moderate-to-vigorous physical activity; VPA, vigorous physical activity; MSA, muscle-strengthening activities; SES, socioeconomic status.

For Hypothesis 1, the measurement model exhibited an acceptable model fit as follows: χ^2^_scaled_ = 6315.84, *df* = 50, *p* < 0.001, CFI_scaled_ = 0.992, TLI_scaled_ = 0.997, RMSEA_scaled_ = 0.048 (90% CI [0.047, 0.049]), SRMR = 0.027). Factor loadings for the anxiety indicators ranged from 0.71 to 0.91, supporting the conceptualization of anxiety symptoms as a latent construct. Table 4 and Figure 3 display the results of the inverse association between PA and anxiety symptoms. Specifically, compared to the reference group (0–2 days/week), both the 3–4 days/week and ≥ 5 days/week categories of MVPA, VPA, and MSA were negatively associated with anxiety symptoms (βs ranging from −0.021 to −0.057, *p* < 0.01). Collectively, these PA variables accounted for 1% of the variance in anxiety symptoms (*R*^2^ = 0.01) in this baseline unadjusted model without sociodemographic covariates, which were addressed in the subsequent analyses for Hypothesis 2.

**Table 4:** Effects of PA frequency across PA types on anxiety symptoms (Reference frequency: 0–2 days).

Variable	*β*	*SE*	95% CI [lower, upper]	*z*	*p*
MVPA (ref: 0–2 days)					
3–4 days	−0.021	0.012	[−0.068, −0.022]	−3.859	< 0.001
≥ 5 days	−0.020	0.015	[−0.077, −0.016]	−2.988	0.003
VPA (ref: 0–2 days)					
3–4 days	−0.032	0.012	[−0.090, −0.044]	−5.813	< 0.001
≥ 5 days	−0.057	0.015	[−0.160, −0.100]	−8.455	< 0.001
MSA (ref: 0–2 days)					
3–4 days	−0.030	0.013	[−0.104, −0.052]	−5.871	< 0.001
≥ 5 days	−0.028	0.018	[−0.134, −0.064]	−5.471	< 0.001

*β*, standardized regression coefficient; CI, confidence interval; SE, standard error; z, z-statistic; *p*, *p*-value; MVPA, moderate-to-vigorous physical activity; VPA, vigorous physical activity; MSA, muscle-strengthening activities; ref, reference category.

**Figure 3:**
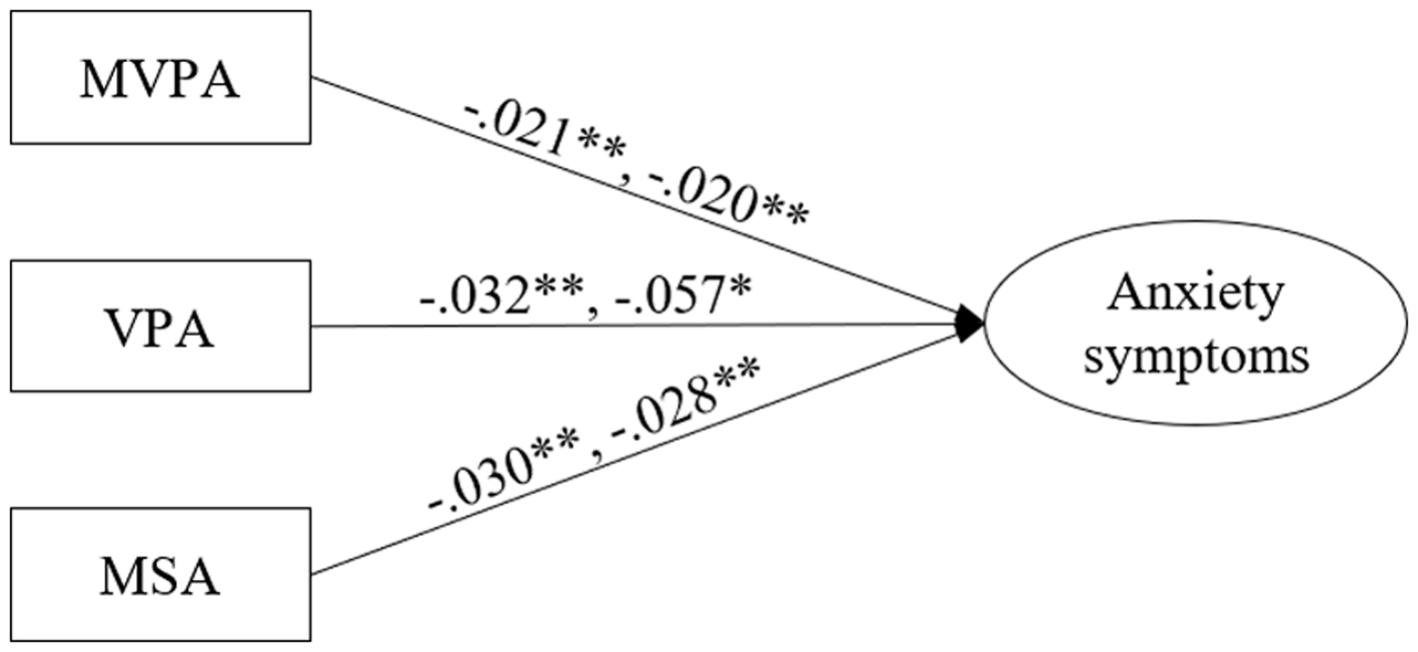
Results of the association between PA and anxiety symptoms. *p < 0.01, **p < 0.001, The first and second values represent standardized regression coefficients (βs) of 3–4 days/week and for ≥ 5 days/week of PA, respectively, compared to the reference group (0–2 days/week).

For Hypothesis 2, the results of the multi-group CFAs demonstrated that all levels of invariance were fully established. As seen in Tables 5, 6, the changes in CFI_scaled_ and RMSEA_scaled_ between nested models (i.e., configural, metric, scalar models) were consistently ≤ 0.001 and ≤ 0.015, respectively, confirming that the latent variable (i.e., anxiety symptoms) is equivalent across gender, school level, and SES as well as across the pairwise comparisons among all SES subgroups (27). Table 7 displays the results of the Satorra–Bentler scaled Δχ^2^_scaled_ revealing significant differences between the baseline (scalar) and path-constrained models across gender (Δχ^2^_scaled_ = 36.60, Δ*df* = 9, *p* < 0.001), school level (Δχ^2^_scaled_ = 79.96, Δ*df =* 9, *p* < 0.001), and SES (Δχ^2^_scaled_ = 48.97, Δ*df =* 16, *p* < 0.001). These results indicate that the structural relationships between PA and anxiety symptoms significantly vary across these sociodemographic factors, leading to a detailed comparison of specific path coefficients.

**Table 5:** Measurement invariance across sociodemographic factors.

Moderator	Model	χ^2^_scaled_	*df*	CFI_scaled_	ΔCFI_scaled_	TLI_scaled_	RMSEA_scaled_	ΔRMSEA_scaled_	SRMR
Gender	Configural	7378.98	136	0.99		0.997	0.044		0.026
	Metric	6620.12	142	0.991	0.001	0.997	0.041	−0.003	0.026
	Scalar	7455.52	155	0.99	−0.001	0.997	0.042	0.001	0.026
School level	Configural	8320.92	136	0.989		0.997	0.047		0.026
	Metric	7244.38	142	0.991	0.002	0.997	0.043	−0.004	0.029
	Scalar	7826.35	155	0.99	−0.001	0.997	0.043	0	0.026
SES	Configural	9105.89	186	0.988		0.996	0.051		0.026
	Metric	7682.88	198	0.99	0.002	0.997	0.046	−0.005	0.027
	Scalar	8159.69	224	0.989	−0.001	0.997	0.044	−0.002	0.026

χ^2^_scaled_, scaled chi-square test statistic; df, degrees of freedom; CFI_scaled_, scaled comparative fit index; ΔCFI_scaled_, change in scaled comparative fit index; TLI_scaled_, scaled Tucker–Lewis index; RMSEA_scaled_, root mean square error of approximation; ΔRMSEA_scaled_, change in scaled root mean square error of approximation; SRMR_scaled_, standardized root mean square residual; SES, socioeconomic status.

**Table 6:** Measurement invariance for pairwise comparison across socioeconomic status subgroups.

Moderator	Model	χ^2^_scaled_	*df*_scaled_	CFI_scaled_	ΔCFI_scaled_	TLI_scaled_	RMSEA_scaled_	ΔRMSEA_scaled_	SRMR
High vs. Middle SES	Configural	8561.92	124	0.988		0.996	0.050		0.026
	Metric	7495.31	130	0.990	+0.002	0.997	0.046	−0.004	0.027
	Scalar	7574.83	143	0.990	0.000	0.997	0.044	−0.002	0.026
High vs. Low SES	Configural	3954.27	124	0.988		0.996	0.050		0.025
	Metric	3452.80	130	0.990	+0.002	0.997	0.046	−0.004	0.026
	Scalar	3807.58	143	0.989	−0.001	0.997	0.046	0.000	0.025
Middle vs. Low SES	Configural	5534.46	124	0.987		0.996	0.053		0.026
	Metric	4843.56	130	0.989	+0.002	0.996	0.048	−0.005	0.027
	Scalar	5288.58	143	0.988	−0.001	0.996	0.048	0.000	0.026

χ^2^_scaled_, scaled chi-square test statistic; df, degrees of freedom; CFI_scaled_, scaled comparative fit index; ΔCFI_scaled_, change in scaled comparative fit index; TLI_scaled_, scaled Tucker–Lewis index; RMSEA_scaled_, root mean square error of approximation; ΔRMSEA_scaled_, change in scaled root mean square error of approximation; SRMR_scaled_, standardized root mean square residual; SES, socioeconomic status.

**Table 7:** Model comparison for testing moderating effects of sociodemographic factors.

Moderator	Model	χ^2^_scaled_	*df*	Δχ^2^_scaled_	Δ*df*	*p*
Gender	Baseline model (scalar)	7455.52	155			
	Path-constrained model	7801.42	164	36.601	9	< 0.001
School Level	Baseline model (scalar)	7826.35	155			
	Path-constrained model	7906.31	164	79.96	9	< 0.001
SES	Baseline model (scalar)	8159.69	224			
	Path-constrained model	8208.66	240	48.97	16	< 0.001

χ^2^, chi-square test statistic; df, degrees of freedom; Δχ^2^, Satorra–Bentler scaled difference in chi-square test statistic; Δdf, difference in degrees of freedom; *p*, *p*-value; SES, socioeconomic status.

Table 8 presents the gender-specific path coefficients of the effects of PA frequency across PA types on anxiety symptoms. Specifically, VPA emerged as a more consistent predictor for males than for females. For males, 3–4 days/week (*β* = −0.073, *p* < 0.001) and ≥ 5 days/week (*β* = −0.094, *p* < 0.001) of VPA significantly negatively predicted anxiety. In contrast, for females, only ≥ 5 days/week of VPA was significantly associated with reduced anxiety symptoms (*β* = −0.056, *p* = 0.030). Regarding MVPA, there was no significant association in males; however, for females, engaging in ≥ 5 days/week was linked to higher anxiety levels (*β* = 0.055, *p* = 0.033). For all genders, MSA did not significantly contribute to anxiety symptoms (*p* > 0.05). In terms of sociodemographic covariates, high school attendance predicted lower anxiety symptoms for males (*β* = −0.033, *p* < 0.001), but was not a significant factor for females (*p* = 0.055). Higher levels of SES were significant predictors of increased anxiety symptoms for both groups, although the effect was slightly more pronounced in females (*β* = 0.063 for middle SES; *β* = 0.088 for high SES) than in males (*β* = 0.040 for middle SES; *β* = 0.074 for high SES). The final model including all PA frequencies across PA types as well as the remaining demographic covariates, such as school level and SES, accounted for 1.0 and 1.1% of the variance in anxiety symptoms for males and females, respectively.

**Table 8:** Path coefficients of the effects of PA frequency across PA types on anxiety symptoms by gender (reference frequency: 0–2 days).

Independent variable	Male			Female		
	*β*	95% CI	*p*	*β*	95% CI	*p*
MVPA (ref: 0–2 days)						
3–4 days	−0.002	[−0.038, 0.033]	0.901	−0.005	[−0.039, 0.028]	0.754
≥ 5 days	−0.012	[−0.055, 0.032]	0.592	0.055	[0.005, 0.105]	0.033
VPA (ref: 0–2 days)						
3–4 days	−0.073	[−0.109, −0.036]	< 0.001	−0.016	[−0.048, 0.017]	0.343
≥ 5 days	−0.094	[−0.137, −0.050]	< 0.001	−0.056	[−0.106, −0.005]	0.030
MSA (ref: 0–2 days)						
3–4 days	−0.022	[−0.057, 0.013]	0.225	0.015	[−0.033, 0.062]	0.545
≥ 5 days	−0.032	[−0.078, 0.013]	0.162	−0.042	[−0.119, 0.034]	0.277
School level (ref: Middle school)						
High school	0.033	[0.010, 0.056]	< 0.001	−0.013	[−0.026, 0.000]	0.055
SES (ref: Low SES)						
Middle SES	0.040	[0.017, 0.063]	< 0.001	0.063	[0.041, 0.086]	< 0.001
High SES	0.074	[0.051, 0.097]	< 0.001	0.088	[0.065, 0.111]	< 0.001

*β*, standardized regression coefficient; *p*, *p*-value; MVPA, moderate-to-vigorous physical activity; VPA, vigorous physical activity; MSA, muscle-strengthening activities; SES, socioeconomic status; ref, reference category.

Table 9 presents the path coefficients of the effects of PA frequency across PA types on anxiety symptoms, moderated by school level. The multi-group analysis revealed that the protective effects of PA against anxiety symptoms were more diverse for high school students compared to middle school students. Specifically, VPA was significantly associated with lower anxiety levels in both groups, but the significant frequency differed between groups. For middle school students, a significant adverse effect was observed only when engaging in VPA for ≥ 5 days/week (*β* = −0.029, *p* = 0.001). In contrast, for high school students, both 3–4 days/week (*β* = −0.022, *p* = 0.007) and ≥ 5 days/week (*β* = −0.029, *p* = 0.004) of VPA significantly predicted lower anxiety symptoms. In addition, while the MSA frequencies exhibited no significant associations with anxiety symptoms among middle school students, a significant association was observed between ≥ 5 days/week of MSA and lower anxiety symptoms for high school students when performed ≥ 5 days/week (*β* = −0.023, *p* = 0.002). For MVPA, no significant path coefficients were found at either school level (*p* > 0.05). In terms of sociodemographic covariates, both females and higher SES were robust predictors of increased anxiety across both groups (*p* < 0.001). However, the impact of SES appeared to be more pronounced in middle school students than in high school students. The proposed model including all PA frequencies across PA types as well as the remaining demographic covariates, such as gender and SES, explained a higher proportion of variance in anxiety symptoms for middle school students (*R*^2^ = 0.061) than for high school students (*R*^2^ = 0.037).

**Table 9:** Path coefficient of the effects of PA frequency across PA types on anxiety symptoms by school level (reference frequency: 0–2 days).

Independent variable	Middle school			High school		
	*β*	95% CI	*p*	*β*	95% CI	*p*
MVPA (ref: 0–2 days)						
3–4 days	0.004	[−0.009, 0.017]	0.547	−0.004	[−0.019, 0.011]	0.604
≥ 5 days	0.012	[−0.005, 0.029]	0.169	−0.005	[−0.025, 0.015]	0.649
VPA (ref: 0–2 days)						
3–4 days	−0.013	[−0.027, 0.001]	0.073	−0.022	[−0.038, −0.006]	0.007
≥ 5 days	−0.029	[−0.047, −0.011]	0.001	−0.029	[−0.049, −0.009]	0.004
MSA (ref: 0–2 days)						
3–4 days	0.003	[−0.012, 0.018]	0.695	−0.010	[−0.025, 0.005]	0.168
≥ 5 days	0.003	[−0.013, 0.019]	0.645	−0.023	[−0.038, −0.008]	0.002
Gender (ref: Male)						
Female	0.217	[0.198, 0.236]	< 0.001	0.154	[0.139, 0.169]	< 0.001
SES (ref: Low SES)						
Middle SES	0.070	[0.048, 0.092]	< 0.001	0.028	[0.012, 0.044]	< 0.001
High SES	0.086	[0.064, 0.108]	< 0.001	0.072	[0.056, 0.088]	< 0.001

*β*, standardized regression coefficient; CI, confidence interval; *p*, *p*-value; MVPA, moderate-to-vigorous physical activity; VPA, vigorous physical activity; MSA, muscle-strengthening activities; SES, socioeconomic status; ref, reference category.

Table 10 presents the results of the multi-group analysis moderated by SES. The results indicated that only in the high SES group, performing MVPA ≥ 5 days/week was significantly associated with increased anxiety symptoms (*β* = 0.111, *p* = 0.011); there was no significant association in other groups (i.e., low and middle SES). Only ≥ 5 days/week of VPA was significant for the low SES group (*β* = −0.024, *p* = 0.021). In the middle SES group, both 3–4 days/week (*β* = −0.024, *p* = 0.001) and ≥ 5 days/week (*β* = −0.030, *p* = 0.001) of VPA were effective. Notably, in the high SES group, engaging in VPA ≥ 5 days/week showed the most potent protective effect observed in this study (*β* = −0.124, *p* = 0.005). MSA was a significant predictor only for the Low SES group at ≥ 5 days/week (*β* = −0.015, *p* = 0.048). Regarding covariates, females were a consistent and strong predictor of higher anxiety symptoms across all SES levels (*p* < 0.001). In addition, high school attendance was associated with higher anxiety in the low SES group (*β* = 0.036, *p* < 0.001) but predicted lower anxiety in the high SES group (*β* = −0.063, *p* = 0.048). The explanatory power of the model, including all PA frequencies across PA types as well as the remaining demographic covariates such as gender and school level, increased with SES level, with *R*^2^ values of 0.038, 0.043, and 0.058 for the low, middle, and high SES groups, respectively.

**Table 10:** Path coefficient of the effects of PA frequency across PA types on anxiety symptoms by SES (reference frequency: 0–2 days).

Independent variable	Low SES			Middle SES			High SES		
	*β*	95% CI	*p*	*β*	95% CI	*p*	*β*	95% CI	*p*
MVPA (ref: 0–2 days)									
3–4 days	−0.011	[−0.029, 0.007]	0.202	0.005	[−0.009, 0.019]	0.471	0.058	[−0.015, 0.131]	0.122
≥ 5 days	−0.004	[−0.025, 0.017]	0.692	0.004	[−0.013, 0.021]	0.634	0.111	[0.025, 0.197]	0.011
VPA (ref: 0–2 days)									
3–4 days	−0.008	[−0.027, 0.011]	0.381	−0.024	[−0.038, −0.010]	0.001	−0.030	[−0.103, 0.043]	0.420
≥ 5 days	−0.024	[−0.045, −0.003]	0.021	−0.030	[−0.048, −0.012]	0.001	−0.124	[−0.210, −0.038]	0.005
MSA (ref: 0–2 days)									
3–4 days	−0.008	[−0.023, 0.007]	0.303	0.001	[−0.014, 0.016]	0.929	−0.009	[−0.075, 0.057]	0.780
≥ 5 days	−0.015	[−0.03, −0.001]	0.048	−0.003	[−0.016, 0.010]	0.668	−0.023	[−0.088, 0.042]	0.489
Gender (ref: Male)									
Female	0.175	[0.122, 0.228]	< 0.001	0.198	[0.183, 0.213]	< 0.001	0.201	[0.180, 0.222]	< 0.001
School level (ref: Middle school)									
High school	0.036	[0.015, 0.057]	< 0.001	−0.008	[−0.020, 0.004]	0.204	−0.063	[−0.125, −0.001]	0.048

*β*, standardized regression coefficient; CI, confidence interval; *p*, *p*-value; MVPA, moderate-to-vigorous physical activity; VPA, vigorous physical activity; MSA, muscle-strengthening activities; SES, socioeconomic status; ref, reference category.

## 4 Discussion

The purpose of this study was to determine the associations between types/intensities and frequencies of PA and anxiety symptoms across sociodemographic factors using a population sample of Korean adolescents. The study tested two hypotheses of the associations between different types/intensities and frequencies of PA and anxiety symptoms (Hypothesis 1) and the associations across gender, school level, and SES (Hypothesis 2). Multiple SEM analyses revealed that all PA types/intensities (i.e., MVPA, VPA, MSA) and frequencies (i.e., 0–2 days, 3–4 days, and 5 days per week) were negatively associated. The associations significantly differed across gender, school level, and SES, suggesting that subgroups of these sociodemographic factors played different roles in the associations. There were also direct associations between sociodemographic factors and anxiety. These findings suggest that PA may be negatively associated with anxiety symptoms regarding different types/intensities and frequencies of PA in the context adolescents encounter. Nevertheless, caution must be taken when interpreting these findings due to the significant but small coefficients. Although the associations between PA frequency and anxiety symptoms reached statistical significance, they may be attributed to the substantial statistical power of this large-scale population sample (*n* = 54,653). In addition, the practical effect size in the overall model was modest, accounting for approximately 1% of the total variance in anxiety (*R^2^* = 0.01). This modest explanatory power underscores that adolescents’ anxiety symptoms are a complex, multifactorial phenomenon that may be largely driven by different determinants, such as academic pressures, sleep deficiency, social relationships, and familial environments other than PA. Therefore, while PA is likely to provide meaningful protective effects, it should not be viewed as an isolated panacea; rather, it should be integrated as a complementary lifestyle component within comprehensive adolescent mental-health support systems for healthful living.

### 4.1 The associations between PA and anxiety symptoms

The analyzed data of this study seem to lend empirical support to the protective role of PA against anxiety symptoms among Korean adolescents (2, 4, 10). In the overall model, participation on 3–4 or ≥ 5 days/week in each PA measure was associated with fewer anxiety symptoms than participation on 0–2 days/week (17, 20). These findings imply that while PA is likely to have a protective effect on anxiety symptoms, the specific types/intensities and frequencies of the activity would matter for lower anxiety (4, 10), suggesting potential frequency thresholds for PA: engaging in MVPA and MSA for 3–4 days per week, and VPA for ≥ 5 days per week, although these thresholds should warrant further investigation in longitudinal and intervention studies. These results are consistent with the possibility that PA supports adolescent mental health, while the small coefficients and cross-sectional design preclude causal conclusions (1–14). Potential explanations include physiological adaptations in stress-response regulation and psychological processes such as increased self-efficacy and opportunities to interrupt persistent worry (9). Different activities may provide some of these shared experiences, which could help explain why inverse associations were observed across all three PA measures rather than for a single activity type.

The social context of participation may also be relevant. PA can provide opportunities for peer interaction, perceived competence, and time away from academic demands (21). Empirical evidence from Korean adolescents further indicates that engaging in physical activity serves as a key strategy to cope with academic stress (2, 10). These findings provide a contextually relevant explanation: for some Korean adolescents, PA may function as a valued break from stressful routines rather than solely as physical exertion. However, the current study did not measure academic stress, activity setting, social support, or participation motives as mediating mechanisms. These explanations should therefore be considered hypotheses for future research rather than demonstrated pathways.

### 4.2 The associations between PA and anxiety symptoms across sociodemographic factors

The results of Satorra–Bentler scaled Δχ^2^ tests and multi-group SEM analyses demonstrated that the associations between types/intensities and frequencies of PA and anxiety symptoms significantly differed across gender, school level, and SES. At least 3 days/week of VPA and ≥ 5 days/week of MSA appeared to have a protective effect compared to participation for 0–2 days. However, a contrasting pattern was observed for the MVPA frequency, where ≥ 5 days/week of MVPA was positively associated with anxiety symptoms across certain sociodemographic factors, compared with the reference group (MVPA for 0–2 days).

For VPA, participation for 3–4 days per week was associated with lower anxiety in specific subgroups, including males, high school students, and those with middle SES. The association was also observed when performing VPA for ≥ 5 days per week across all subgroups of gender (males and females), school level (middle and high school), and SES (low, middle, and high SES), compared with 0–2 days. Engaging in VPA for 3–4 days per week may be sufficient to confer emotional or psychological stability in males, high school students, and middle SES adolescents (14). In contrast, for females, middle school students, and adolescents with low and high SES, VPA for 3–4 days per week might not be sufficient for lower anxiety symptoms. However, it should be noted that interpreting these subgroup variations requires caution. Rather than assuming that all subgroups require the same magnitude of symptom reduction or a higher frequency, these patterns may reflect variations in baseline anxiety levels across groups or differential physiological and psychological responses to vigorous activity. Although Chekroud et al. (17) concluded that the optimal frequency of VPA was 3–5 days per week for lower anxiety symptoms across various sociodemographic factors, such as gender, age, and SES, among 1.2 million US individuals, the nuanced variations observed here underscore a potential context-specific nature of VPA for lower anxiety, which could lead to the inconsistency unexplained in this study, highlighting the need for further research to examine these differences.

Regarding MSA, this study found that compared with 0–2 days/week of the activity, ≥ 5 days was associated with lower anxiety, specifically among high school students and those from low SES backgrounds; however, no significant association was observed among other subgroups. These findings imply that both a high-frequency threshold and subgroup-specific conditions are needed for the anxiolytic benefits of MSA to emerge in Korean adolescents. These findings are partially consistent with previous studies reporting an inverse association between MSA and anxiety. When analyzed separately by gender, Wang et al. (33) found that more frequent MSA engagement declined mental health problems, such as depression symptoms in both females and males. Shannon et al. (34) suggested that at least 3 days or more of MSA is the optimal frequency threshold for reducing anxiety symptoms among adolescents in Ireland. This inconsistency of the MSA frequency implies that the MSA frequency for lower anxiety symptoms is not uniformly observed across all subgroups of gender, school level, and SES, but rather emerges only beyond a higher frequency threshold (i.e., ≥ 5 days/week) within specific sub-populations, such as high school students and those with low SES among Korean adolescents. Although speculative, one possible explanation is that Korean high school students and adolescents with low SES might experience psychological benefits from frequent MSA participation. According to Ho et al. (35), PA perceived as structured and goal-oriented, such as MSA, can foster positive emotional experiences. In addition, Anderson and Shivakumar (9) suggested that engagement in MSA is likely to decrease anxiety arousal by facilitating self-efficacy. High school students and adolescents from low SES backgrounds in this study might experience a sense of self-mastery, physical competence, and perceived achievement when involved in challenging activities, such as MSA, for ≥ 5 days per week, leading to lower anxiety symptoms. Consequently, while previous studies have emphasized the general benefits of frequent MSA across populations, the present study extends the literature by highlighting a higher MSA frequency associated with positive psychological experiences, such as self-efficacy, among high school students and/or those with lower SES. However, as they were not directly tested in the current study, such interpretations remain hypothetical, calling for further clarification of the mechanisms linking MSA, self-efficacy, and anxiety among different populations.

An interesting finding was that MVPA participation on ≥ 5 days/week was positively associated with anxiety symptoms among females and adolescents in the high-SES group. This association should not be interpreted as evidence that frequent MVPA causes anxiety. Indeed, while PA is, in general, recognized for its mental health benefits, prior literature has indicated that its association with anxiety symptoms is not always uniform or linear, with several studies reporting null or inconsistent findings across adolescent populations (2, 5, 10, 15, 19, 20). Building upon these inconsistent patterns across studies, several alternative explanations should be considered. First, the association between the MVPA frequency and anxiety symptoms might be reversed. Rather than high-frequency MVPA directly driving up anxiety symptoms, adolescents who experienced higher baseline anxiety symptoms or underlying psychological distress may participate in frequent MVPA as a means of managing their anxiety symptoms or facilitating mental health benefits. This bidirectional vulnerability cannot be ruled out in cross-sectional research designs. As highlighted in longitudinal studies and comprehensive reviews (9, 12), psychological distress or anxiety symptoms can influence subsequent PA levels; establishing a definitive direction of causality remains methodologically challenging in the current cross-sectional research design. Second, measurement error and information bias inherent in self-reported questionnaires may have contributed to these positive associations. Self-reported PA is inherently limited by recall bias, social desirability, or misclassification, where adolescents may over- or under-report their actual activity frequencies, introducing substantial measurement error into the observed effect sizes (7, 10–15, 20). Finally, unmeasured confounding variables and specific contextual factors appear to be a critical contributor to the association. Factors such as academic pressure, performance stress, overtraining, or unmeasured socioeconomic and personality characteristics may interact with frequent MVPA in different sociodemographic subgroups (e.g., females, adolescents with high SES). Participating in frequent MVPA can be conditionally protective; when adolescents experience excessive stress, perfectionism, or performance demands, frequent MVPA can reflect or even exacerbate underlying distress, producing complex, non-linear, or mixed patterns rather than straightforward protective effects (36). Consequently, while previous studies generally suggest inverse relationships between PA and anxiety symptoms (4, 9, 11, 13, 19, 20), the positive associations observed in specific subgroups are not conclusive. Future longitudinal and experimental research is warranted to test these alternative hypotheses directly and to clarify the complex mechanisms linking MVPA frequency and anxiety symptoms across diverse demographic groups.

### 4.3 Strengths and limitations

This study has several strengths. First, using a large-scale, nationally representative sample of Korean adolescents from KYRBS, this study provides broad descriptive insights into the patterns of PA relevant to anxiety symptoms among middle and high school students in South Korea. Second, this study provided a nuanced understanding of the associations between PA and anxiety symptoms through the categorization of PA into specific types/intensities and frequencies rather than treating it as a monolithic construct. This approach allows for the identification of complex associations with anxiety across different sociodemographic factors, including gender, school level, and socioeconomic status. Lastly, this study employed a structured modeling approach to examine the associations between these variables while accounting for key covariates, offering useful foundational data for future hypothesis testing.

There were also several limitations that should be acknowledged regarding research design, measurement, and statistical analysis. First, this study adopted a cross-sectional design, which limits establishing a definitive causal relationship between PA and anxiety. In addition, the findings may be limited, as the data were uniquely situated within the South Korean context. Second, each type/intensity of PA was assessed using a single self-reported item from the KYRBS, with duration specified only for MVPA (≥ 60 min) but not for VPA and MSA, which may introduce recall bias and potentially affect the reliability and validity of the measured activity levels. Such self-reported measures are inherently susceptible to information bias, including recall bias and social desirability bias, which may affect the precision of the activity levels. Additionally, PA frequencies were categorized into three groups (0–2, 3–4, and ≥ 5 days) to ensure model stability across subgroups, but this categorization might influence the observed associations between PA and anxiety symptoms. It is suggested that future research incorporate objective, device-based measures or conduct sensitivity analyses to evaluate potential threshold effects with greater precision. Third, the potential for unmeasured confounding variables, such as genetics, family environment, academic stress, and relationships with family members and peers, must be acknowledged, as these contextual factors could account for the observed associations, particularly the unexpected positive findings in specific subgroups. Fourth, while complex sampling weights were not directly incorporated into the analyses, key stratification design factors were conditioned as covariates and grouping variables to minimize selection bias (30). Nevertheless, future studies accounting for the complex sampling structure are recommended to perform sensitivity analyses comparing weighted and unweighted models. Finally, while measurement invariance was supported by conservative cutoffs (ΔCFI_scaled_ ≤ 0.010, ΔRMSEA_scaled_ ≤ 0.015), the potential sensitivity of goodness-of-fit indices to large sample sizes remains a methodological limitation.

## 5 Conclusion

This study demonstrated the associations between PA types/intensities and frequencies and anxiety symptoms among South Korean adolescents. The findings indicated that participation on 3–4 and ≥ 5 days/week was generally associated with fewer anxiety symptoms in the overall model, but the patterns were not uniform across gender, school level, and SES, underscoring that adolescent mental health interventions cannot rely on a uniform approach. From a public health and clinical perspective, these findings suggest that education and health authorities design flexible, context-sensitive PA programs that accommodate diverse sociodemographic backgrounds and needs rather than assuming identical benefits for all groups. Such programs should complement, rather than replace, appropriate mental-health assessment and support, and participation and mental-health outcomes should be evaluated across sociodemographic groups. The positive associations observed in selected subgroups should prompt further investigation rather than recommendations to reduce PA. However, given the cross-sectional design and small effect sizes, these findings need to be conducted to verify the causal relationship between PA and anxiety symptoms through prospective studies and/or interventions. In addition, future research should prioritize longitudinal research designs, objective device-based PA measurements, and mechanism-focused investigations to establish definitive, causality-based PA recommendations for adolescent mental health.

## Data Availability

Publicly available datasets were analyzed in this study. This data can be found at: https://www.kdca.go.kr/yhs.
